# Examining the effectiveness of gamification as a tool promoting teaching and learning in educational settings: a meta-analysis

**DOI:** 10.3389/fpsyg.2023.1253549

**Published:** 2023-10-09

**Authors:** Minzi Li, Siyu Ma, Yuyang Shi

**Affiliations:** ^1^Center for Linguistics and Applied Linguistics, Guangdong University of Foreign Studies, Guangzhou, China; ^2^Law School, Peking University, Beijing, China

**Keywords:** gamification, learning outcomes, educational settings, meta-analysis, design principles, gameful experience, learning environment

## Abstract

The integration of gamification into educational settings has gained recognition for its potential to boost student motivation, engagement, interest, and learning outcomes. Despite its popularity, research on gamification has produced mixed results regarding student learning outcomes. This meta-analysis aims to synthesize the existing empirical evidence on the effectiveness of gamification as a tool for promoting teaching and learning in educational settings. Forty-one studies with 49 independent samples involving more than 5,071 participants were included in our analysis. Results from random effects models showed an overall significant large effect size (*g* = 0.822 [0.567 to 1.078]). The research performed the moderator analysis to scrutinize the effects of a number of factors on the relationship between gamification and student learning outcomes. The study uncovered significant moderating effects for user type, educational discipline, design principles for educational gamification, duration of “gameful” experience, and learning environment. However, measurement of student outcomes and publication type did not appear to have any significant moderating effect. Those findings hold important implications for improving and implementing gamification to promote teaching and learning in future research.

## 1. Introduction

Over the past 40 years, the field of educational technology research has undergone significant development, evolving from a specialized niche area to a prominent subfield within education. Alongside this evolution, the focus of research has shifted from an almost sole emphasis on the effect of technology on learning outcomes to a broader examination of different aspects of educational technology (e.g., usage, evaluation). One such aspect that has garnered increasing attention in recent years is gamification (Domínguez et al., 2013; Huang et al., 2020). Coined in 2008 (Marczewski, 2013), gamification refers to using elements from games in non-gaming environments (Deterding et al., 2011; Garland, 2015). It is important to distinguish gamification from game-based learning, as the former as a broader concept only utilizes components of games in real-world situations, while the latter employs full-featured games to deliver skill or knowledge (Kapp, 2012; Wijaya et al., 2022). Gamification has found widespread application in various domains (Caponetto et al., 2014), including business (Moradian et al., 2014), math (Yıldırım, 2017), and science (Chen et al., 2018). In recent years, there has been a notable increase in studies exploring gamification in educational contexts (Seaborn and Fels, 2015). The key challenges facing modern education are often attributed to students’ lack of engagement and motivation to actively participate in the learning process. One potential solution to address this issue involves introducing rewards and recognition for students’ efforts and accomplishments, thereby fostering increased motivation and participation. This approach is rooted in the utilization of game elements to enhance the learning experience (Kiryakova et al., 2014). In education, gamification is not a standalone product, but rather a creative and structured process of incorporating game elements into educational settings with the aim of motivating learner behavior and promoting academic achievement (Educause, 2011; Jacobs, 2013). There is a growing body of evidence suggesting that gamification has become widely recognized as an effective tool in promoting learning outcomes in various educational settings (Groening and Binnewies, 2019; Lopez and Tucker, 2019).

Research has shown many advantages of gamification in educational contexts. For example, recent studies have validated the potential of gamification to improve student motivation, engagement, and interaction in education, while allowing them to immerse themselves in experiential learning (Lopez and Tucker, 2019). This is particularly important in the context of psychology, where motivation is seen as the driving force behind the behavior with a dynamic relationship between internal and intrinsic forces and affective processes leading to personal, social, and psychological well-being (Sailer et al., 2017). Gamification was found to increase extrinsic and intrinsic motivation by getting learners involved in tasks through ludic activities (Buckley and Doyle, 2016), and this is particularly evident in the educational context, where gamification promotes student participation in the classroom and stimulates direct interaction between students and teachers (Alsawaier, 2018). The relationship between gamification and learning in education is further explained by theories such as the theory of gamified learning, which suggests that gamification has the potential to positively impact both instructional content and learning outcomes (Landers, 2014). Manzano-León et al. (2021) also highlighted gamification as a highly effective tool in promoting the development of curricular, and cognitive competencies.

Despite the growing popularity and potential benefit of gamification, its effectiveness of it in improving academic performance remains uncertain. The previous empirical literature has produced mixed results. Some studies have reported positive effects of gamification with varying effect sizes (Chen and Chiu, 2016; Homer et al., 2018), while others have found no effects (Rachels and Rockinson-Szapkiw, 2018). These conflicting results make it difficult to draw conclusions about the effectiveness of gamification in enhancing learning outcomes. In order to shed more light on this issue, efforts need to be made to quantify the impact of gamification on student achievement in education. In this research, we conduct a meta-analytic study that thoroughly explores the issue through moderator analysis. The analysis includes a number of variables: user type, educational discipline, design principles for educational gamification, duration of “gameful” experience, learning environment, measurement of student outcomes and publication type. The subsequent section of this paper will delve into each of these moderators in detail.

### 1.1. Moderator factors

#### 1.1.1. User type

The variable, user type, may generate varying results (Grivokostopoulou et al., 2019; Eltahir et al., 2021). Users with a higher education level were found to achieve better learning outcomes (Lister, 2015; Kim and Castelli, 2021) as they were found of higher engagement towards the gamified intervention (Wang et al., 2009; Jahnke, 2010) and stronger sensitivity to the social modeling (Morris and Venkatesh, 2000). However, other studies found that the effect of gamification design on students’ academic performance may not vary based on their level of schooling (Morris and Venkatesh, 2000). Given these conflicting results, we decided to examine the user type as a potential moderator factor in our study.

#### 1.1.2. Educational discipline

Gamified learning’s effects on student learning outcomes can vary depending on the discipline. Over the past few years, gamification has garnered interest in various academic fields (Bai et al., 2020). Subhash and Cudney (2018) emphasized how the subject area affects the approach to gamified learning. The computing field has been the primary focus of gamified learning studies, likely due to its ability to facilitate the creation and investigation of gamified applications and platforms (Sun-lin and Chiou, 2017; Tsai et al., 2020). However, there are other educational disciplines like science, language, and communication where gamification can be applicable and feasible (Subhash and Cudney, 2018). To better understand the conditions that influence the effectiveness of gamification, we decided to investigate the educational disciplines (see details in the coding scheme).

#### 1.1.3. Design principles for educational gamification

Design principles for educational gamification is a critical factor in developing effective gamification strategies for achieving positive learning outcomes. Within the gamification research landscape, several widely used approaches, such as the PBL triangle (i.e., the interaction of points, badges, and leaderboards) (Werbach and Hunter, 2015), the gamification design model (Mora et al., 2016), or the framework for agile gamification of E-learning (Wongso et al., 2015) have gained significant recognition. However, despite the inherent strengths of these approaches, their efficiency may be limited when confronted with the intricate and strategic process of gamification (Navarro-Mateos et al., 2021). For instance, the use of gamification elements in educational settings has often been confined to simple point-based systems, commonly referred to as “Pointification,” which fails to fully leverage the potential of gamification (Seaborn and Fels, 2015). Similarly, the player-centered design frameworks can only be applicable to specific domains, leading to a potential gap between the promise of gamification and its current applications in education. Thus, it is imperative to acknowledge the necessity for a reflective and elaborate design process, for which more comprehensive models like the MDA model proposed by Hunicke et al. (2004) come into play (González-Fernández et al., 2022). This framework offers a structured approach to incorporating game elements and facilitates the balanced design principles of game mechanics, dynamics, and esthetics in educational contexts. While initially developed for game design, the MDA framework has gained widespread adoption and application in the field of gamification (Werbach et al., 2012). Furthermore, empirical evidence, exemplified by the study conducted by Cordero-Brito and Mena (2020), solidifies the significance and broad utilization of the MDA model in gamification research. Their comprehensive analysis of gamification articles published between 2011 and 2016 identified the MDA model as the most prevailing instructional design model in the gamification literature. This underscores the importance of delving deeper into the impact of design principles for educational gamification and highlights the usefulness of the MDA system in doing so (Manzano-León et al., 2021). Manzano-León et al. (2021) classified design principles for educational gamification based on the MDA system, enabling the identification of mechanics, dynamics, and esthetics, as well as their potential combinations, for possible incorporation into gamification strategies. By using the MDA system as a guide, educators or instructors can design and implement gamification in education more effectively, resulting in enhanced student learning outcomes.

#### 1.1.4. Duration of “gameful” experience

The duration of “gameful” experience, which refers to the length of time in which the gamification is implemented, is an essential factor to consider in evaluating the effectiveness of gamification on student achievement. Previous research has yielded conflicting results (Tsay et al., 2018; Mahmud et al., 2020; Mays et al., 2020). Kim and Castelli (2021) suggested that long-term intervention (i.e., 20 weeks) may significantly facilitate learners’ engagement and motivation, resulting in higher academic performance. However, other studies have found that the optimal peak effect of gamified learning is achieved in shorter durations, typically less than a week (Racey et al., 2016; Lei et al., 2022). Therefore, this moderator was introduced in our meta-analysis to examine the effectiveness of gamification in promoting teaching and learning in educational contexts. We categorized “gameful” experience into five groups based on length (see details in the coding scheme).

#### 1.1.5. Learning environment

The success of incorporating gamification in education is heavily influenced by the learning environment in which it is being used. Previous studies have emphasized the significance of learning conditions, whether it be online, offline, or in a hybrid format, in shaping how students interact with gamification and their overall learning outcomes (Huang and Hew, 2015; Ninaus et al., 2015; Stansbury and Earnest, 2016). However, the role of the learning environment as a moderator of the effectiveness of gamification has produced mixed results in previous research. Some studies have reported that certain learning conditions can improve academic achievement, while others have yielded conflicting results (Stansbury and Earnest, 2016; Denny et al., 2018). Therefore, it is essential to identify the optimal learning environment for implementing gamification based on empirical literature to ensure its effectiveness.

#### 1.1.6. Measurement of student outcomes

The implementation of gamification in educational contexts has been increasingly explored as a means to enhance students’ learning outcomes (Ritzhaupt et al., 2021). However, the efficacy of gamification in education has produced inconsistent findings (Huang et al., 2020). Inocencio (2018) attributed this contentiousness to the issue of measurement, as different measures are employed to evaluate student outcomes, making it difficult to discern patterns (Johnson et al., 2016). Academic performance, motivation, and engagement are frequently utilized outcome measures in gamification research (Dichev and Dicheva, 2017; Zainuddin et al., 2020; Rivera and Garden, 2021). Academic performance provides a tangible measure of the effectiveness of gamification in improving students’ knowledge acquisition and application (Rivera and Garden, 2021). Motivation holds importance as it influences students’ attention, persistence, and effort in learning activities (Brophy et al., 2013). Engagement reflects students’ dedication and participation in the subject and various tasks (Segura-Robles et al., 2020). However, there is a lack of uniformity in measurements employed in previous research to measure the outcomes of gamification (Tomaselli et al., 2015; Sailer and Homner, 2020). To address this issue, our objective was to investigate whether the effectiveness of gamification in educational settings remains consistent when evaluated using different measures to assess student outcomes.

### 1.2. Research aims

The present study aims to provide an updated comprehensive synthesis of the current literature on gamification, with a specific focus on assessing the impact of moderator variables on the variability of effect sizes between studies regarding gamification and learning outcomes.

Two research questions have been formulated to achieve the study’s objectives.

(1) What is the overall effect of gamification on students’ learning outcomes?
(2) What factors may moderate the effects of gamification on student learning outcomes?

## 2. Materials and methods

### 2.1. Identifying primary studies

The procedure for locating related primary studies was carried out as follows. First, we conducted a thorough search across academic databases to locate pertinent studies released from 2010 to 2022, including *ACM Digital Library, ERIC, IEEE Xplore, JSTOR, PubMed, ScienceDirect, Scopus, Web of Science and SpringerLink*. For the purpose of this review, two sets of keywords were employed. The first set comprised terms related to gamification, using gamif* to encompass all possible variations of “gamification,” “gamify,” and “gamified learning.” The second set of search terms included terms pertained to learning, course, class, performance, outcomes, education, influence, impact, or effect, which were used in the following search query: gamif* AND (learning OR education OR course OR class OR performance OR outcomes OR influence OR impacts OR effects). In addition to these databases, the same keywords were also used to search Google and Google Scholar.

### 2.2. Inclusion and exclusion criteria

#### 2.2.1. Inclusion criteria

Upon identification of primary studies, the subsequent inclusion and exclusion criteria were utilized to arrive at the final sample of studies.

1. Studies should adhere to the defined concept of gamification as provided in the study.
2. Studies were required to report sufficient statistical data (e.g., means, sample sizes) to make the meta-analytic techniques applicable.
3. The study should specify the target population as students at any educational level within the context of a formal educational setting.
4. Studies were obligated to present at least one comparison of learning outcomes between a class that utilized gamification and one that did not (i.e., a between-subjects research design). Within-subject designs were deleted because they could lead to carry-over effects (Charness et al., 2012).
5. Eligible studies should be reported in English.

#### 2.2.2. Exclusion criteria

1. Studies were excluded from the analysis if their sole focus was on game-based learning.
2. Studies were removed from the analysis if they relied exclusively on students’ self-reported results without objective measures.
3. Studies were disqualified if they did not provide precise details regarding the design principles for educational gamification employed.
4. Studies that were limited to only offering theoretical discussions or non-empirical descriptions of gamification were ruled out from the analysis.

**Figure 1:**
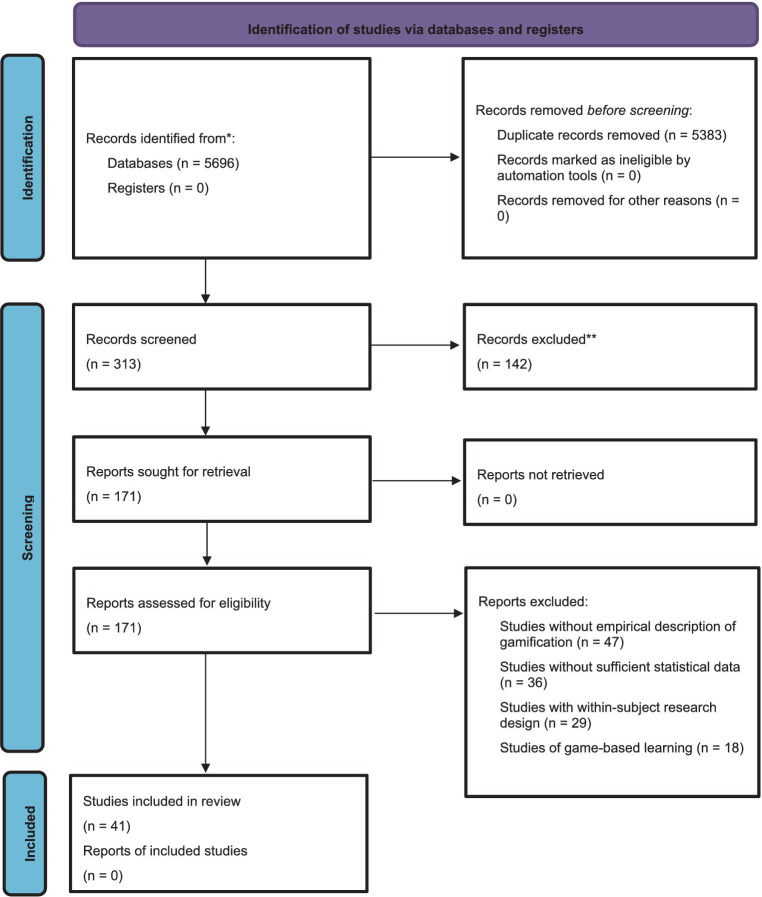
PRISMA (Page et al., 2021) flowchart of the selection process.

The process flowchart for the search, identification, screening, coding and extraction stages in our investigation is presented in Figure 1.

### 2.3. Coding scheme

The codebook, which was developed using the existing literature on gamification, was applied to code the data. The following is a summary of the codebook:

- Author information including author(s) and year of publication (e.g., 2017; 2021).
- Publication type (1-Journal article, 2-Thesis/dissertation, 3-Conference proceeding).
- User type (1-Elementary school user, 2-Secondary school user; 3-Higher education user).
- Educational discipline (1-Science, 2-Math, 3-Engineering/computing, 4-Social science, 5-Business, 6-Others, 7- Not mentioned).
- Design principles for educational gamification (1-Mechanism, 2-Dynamics + Esthetics, 3-Mechanics; 4-Mechanics + Dynamics; 5-Mechanics + Dynamics + Esthetics).
- Duration of “gameful” experience (1-1-week, 2–1 week-1 month, 3–1 month-3 months, 4–3 months-1 semester, 5- > 1 semester)
- Learning environment (1-Online, 2-Offline, 3-Hybrid).
- Measurement of student outcomes (1-Academic performance, 2-Engagement, 3-Motivation).
- Statistical information required to calculate effect sizes (e.g., mean, sample size, standard deviation).

### 2.4. Reliability of coding

In order to perform the task, two coders were trained and instructed on the coding protocol, which was then practiced in seven studies. The results were subsequently discussed and deliberated to ensure that all data was coded adhering to the established coding protocol. The two coders then coded all included qualifying studies independently. The percentage of absolute agreement between the two coders was 96.4%. Any discrepancies were resolved through discussion. The rigorous training and thorough coding process used in this study ensured high inter-coder reliability, thereby increasing the validity and accuracy of the findings. A descriptive summary of the included studies is presented in Table 1 to provide an overview of the research evidence analyzed.

**Table 1:** Quantitative description of included studies.

Study (Year)	Publication type	Participant		User type	Educational discipline	Design principles for educational gamification	Duration of “gameful” experience	Learning environment	Student outcomes
		Treatmentn	Controln						
Alzaid (2018)	Thesis/dissertation	10	10	Higher education user	Social science	Mechanics + Dynamics + Esthetics	1 week–1 month	Hybrid	Academic performance
Bernik et al. (2015)	Conference proceeding	28	27	Higher education user	Engineering/computing	Mechanics + Dynamics + Esthetics	> 1 semester	Hybrid	Academic performance
Bernik et al. (2018)	Conference proceeding	87	95	Higher education user	Engineering/computing	Mechanics + Dynamics + Esthetics	1 week–1 month	Online	Academic performance
Chen and Chiu (2016)	Journal article	28	30	Elementary school user	Engineering/computing	Mechanics + Dynamics	1 month-3 month	Hybrid	Engagement
Chen et al. (2018)	Journal article	44	36	Higher education user	Science	Mechanics + Dynamics + Esthetics	> 1 semester	Online	Academic performance
Chen et al. (2015)	Journal article	51	47	Elementary school user	Engineering/computing	Mechanics + Dynamics	<1 week	Hybrid	Motivation
Cosgrove (2016)	Thesis/dissertation	22	22	Higher education user	Engineering/computing	Mechanics + Dynamics + Esthetics	1 week–1 month	Hybrid	Academic performance
DeLeeuw and Mayer (2011)	Journal article	86	48	Higher education user	999	Mechanics + Dynamics	<1 week	Online	Academic performance
Denny et al. (2018)	Conference proceeding	521	180	Higher education user	Science	Mechanics	3 month–1 semester	Online	Engagement
Eltahir et al. (2021)	Journal article	54	53	Higher education user	Social science	Mechanics + Dynamics + Esthetics	3 month–1 semester	Hybrid	Academic performance
Grivokostopoulou et al. (2019)	Journal article	43	43	Higher education user	Business	Dynamics + Esthetics	1 week–1 month	Online	Academic performance
Hakulinen et al. (2013)	Journal article	142	139	Higher education user	Engineering/computing	Mechanics	3 months–1semester	Online	Engagement
Homer et al. (2018) Study 1	Journal article	18	14	Elementary school user	Social science	Mechanics	1 month–3 month	Hybrid	Academic performance
Homer et al. (2018) Study 2	Journal article	16	16	Elementary school user	Social science	Mechanics	1 month–3 month	Hybrid	Academic performance
Homer et al. (2018) Study 3	Journal article	16	13	Elementary school user	Social science	Mechanics	1 month–3 month	Hybrid	Academic performance
Homer et al. (2018) Study 4	Journal article	13	14	Elementary school user	Social science	Mechanics	1 month–3 month	Hybrid	Academic performance
Hong and Masood (2014)	Journal article	29	31	Secondary school user	999	Mechanics + Dynamics + Esthetics	<1 week	Hybrid	Engagement
Huang and Hew (2015)	Conference proceeding	21	19	Higher education user	Engineering/computing	Mechanics + Dynamics + Esthetics	999	Hybrid	Engagement
Huang et al. (2019)	Journal article	25	23	Higher education user	Social science	Mechanics + Dynamics + Esthetics	1 week–1 month	Hybrid	Engagement
Jo et al. (2018)	Journal article	30	30	Secondary school user	Engineering/computing	Mechanics + Dynamics + Esthetics	1 month–3 months	Hybrid	Engagement
Krause et al. (2015) Study 1	Conference proceeding	67	71	Higher education user	Engineering/computing	Mechanics + Dynamics	1 week–1 month	Online	Engagement
Krause et al. (2015) Study 2	Conference proceeding	67	68	Higher education user	Engineering/computing	Mechanics + Dynamics	1 week–1 month	Online	Engagement
Lam et al. (2018) Study 1	Journal article	22	30	Secondary school user	Social science	Mechanics	1 month–3 month	Hybrid	Academic performance
Lam et al. (2018) Study 2	Journal article	22	20	Secondary school user	Social science	Mechanics	1 month–3 month	Hybrid	Academic performance
Liao et al. (2018)	Journal article	139	136	Higher education user	Social science	Dynamics	> 1 semester	Hybrid	Academic performance
Liu (2016)	Journal article	57	53	Higher education user	Engineering/computing	Mechanics + Dynamics	1 week–1 month	Hybrid	Academic performance
Mahmud et al. (2020)	Journal article	28	20	Higher education user	Science	Mechanics + Dynamics	1 month–3 month	Hybrid	Academic performance
Mays et al. (2020)	Journal article	24	24	Elementary school user	Social science	Mechanics + Dynamics	1 month–3 month	Hybrid	Academic performance
Moradian et al. (2014)	Conference proceeding	9	12	Higher education user	Business	Mechanics + Dynamics	<1 week	Online	Engagement
Ninaus et al. (2015)	Journal article	13	17	Higher education user	999	Mechanics + Dynamics	<1 week	Online	Academic performance
Ortiz-Rojas et al. (2017)	Conference proceeding	50	50	Higher education user	Social science	Mechanics	1 month–3 month	Hybrid	Motivation
Parra-González et al. (2020)	Journal article	35	25	Secondary school user	999	Mechanics	1 week–1 month	Hybrid	Academic performance
Poole et al. (2014)	Journal article	31	23	Higher education user	Business, information system	Mechanics + Dynamics	1 week–1 month	Hybrid	Academic performance
Poondej and Lerdpornkulrat (2016)	Journal article	304	273	Higher education user	Business	Mechanics + Dynamics	999	Hybrid	Engagement
Silpasuwanchai et al. (2016)	Conference proceeding	15	15	Higher education user	999	Mechanics	999	Online	Engagement
Stansbury and Earnest (2016)	Journal article	49	44	Higher education user	Science	Mechanics + Dynamics + Esthetics	> 1 semester	Offline	Academic performance
Star (2015) Study 1	Thesis/dissertation	104	95	Higher education user	Social science	Mechanics + Dynamics + Esthetics	3 month–1 semester	Hybrid	Engagement
Star (2015) Study 2	Thesis/dissertation	95	95	Higher education user	Engineering/computing	Mechanics + Dynamics + Esthetics	3 month–1 semester	Hybrid	Engagement
Sun-Lin and Chiou (2017) Study 1	Journal article	24	24	Elementary school user user	Math	Mechanics	1 week–1 month	Hybrid	Academic performance
Sun-Lin and Chiou (2017) Study 2	Journal article	24	25	Elementary school user user	Math	Mechanics	1 week–1 month	Hybrid	Academic performance
Tsai et al. (2020)	Journal article	17	18	Elementary school user user	Science	Mechanics + Dynamics	1 month–3 month	Hybrid	Academic performance
Tsay et al. (2018)	Journal article	68	68	Higher education user	Business	Mechanics + Dynamics	> 1 semester	Hybrid	Engagement
Turan and Meral (2018)	Journal article	23	23	Secondary school user	Social science	Mechanics + Dynamics + Esthetics	1 week–1 month	Hybrid	Engagement
Turan et al. (2016)	Journal article	46	48	Elementary school user	Engineering/computing	Mechanics + Dynamics	1 month–3 months	Online	Academic performance
White (2020)	Thesis/dissertation	13	17	Higher education user	Others	Mechanics	999	Hybrid	Academic performance
Yıldırım (2017)	Journal article	49	48	Higher education user	Math	Mechanics + Dynamics + Esthetics	3 month–1 semester	Hybrid	Academic performance
Young and Wang (2014) Study 1	Journal article	27	25	Elementary school user	Social science	Mechanics + Dynamics	1 week–1 month	Hybrid	Academic performance
Young and Wang (2014) Study 2	Journal article	26	26	Secondary school user	Social science	Mechanics + Dynamics	1 week–1 month	Hybrid	Academic performance
Zainuddin (2018)	Journal article	27	29	Secondary school user	Science	Mechanics	1 month–3 month	Hybrid	Motivation

### 2.5. Analysis

Comprehensive Meta-Analysis (CMA) version 3.7 was adopted to conduct our analysis (Borenstein et al., 2005). CMA is a commercially available software program that provides a wide array of meta-analytic functions, including the ability to calculate effect sizes, examine moderator effects, and detect publication bias. CMA provides two common models for estimating effect sizes (i.e., the fixed effect model and the random effects model). The fixed effect model assumes a consistent true effect size across all studies, implying that any discrepancies in effect sizes between studies solely arise from sampling errors (Borenstein et al., 2021). The random effects model posits that discrepancies in effect sizes may be due to between-study differences as well as sampling errors. Given our observation of a good proportion of between-study variance, we opted to utilize the random effects model in this study.

This meta-analysis comprised studies carried out in diverse contexts and disciplines and involved different types of users. Moreover, the studies employed a variety of design principles and implemented gamification for varying duration and in diverse learning environments. As a result, we can reasonably assume that a good amount of variance was ascribed to between-study differences. As for study weighting, the random effects model allocates weights to each of the included studies in the analysis employing the inverse of variance method to minimize within-study variance and estimated between-study variance.

To prevent potential bias, multiple effect sizes within one study were combined in the meta-analysis, and each study was limited to one effect size, except for studies with multiple independent samples. Each sample in these studies was considered a separate study, providing unique information to the meta-analysis (Borenstein et al., 2021).

Standardizing effect sizes is a crucial step in any meta-analysis before analyzing data. In this study, we opted to use Hedge’s g as the standardized measure of effect size. This decision was informed by the fact that Hedge’s g is superior to Cohen’s d in tackling bias caused by small sample sizes (Borenstein et al., 2021). After the overall effect size of gamification on learning outcomes was calculated, the heterogeneity of studies was checked by *Q* statistics. The *Q* test compares observed errors to expected sampling errors to determine if differences in effect sizes can be attributed to between-study differences (Lipsey and Wilson, 2001).

To explore the source of heterogeneity, the between-study *Q* statistics were employed in the moderator analysis. *T*^2^ and *I*^2^ calculations were also performed for the estimation of dispersion that resulted from between-study differences. *T*^2^ represents the degree of true heterogeneity while *I*^2^ indicates the dispersion observed in relation to true heterogeneity (Borenstein et al., 2021).

## 3. Results

### 3.1. Overall effect of gamification on student learning outcomes

**Figure 2:**
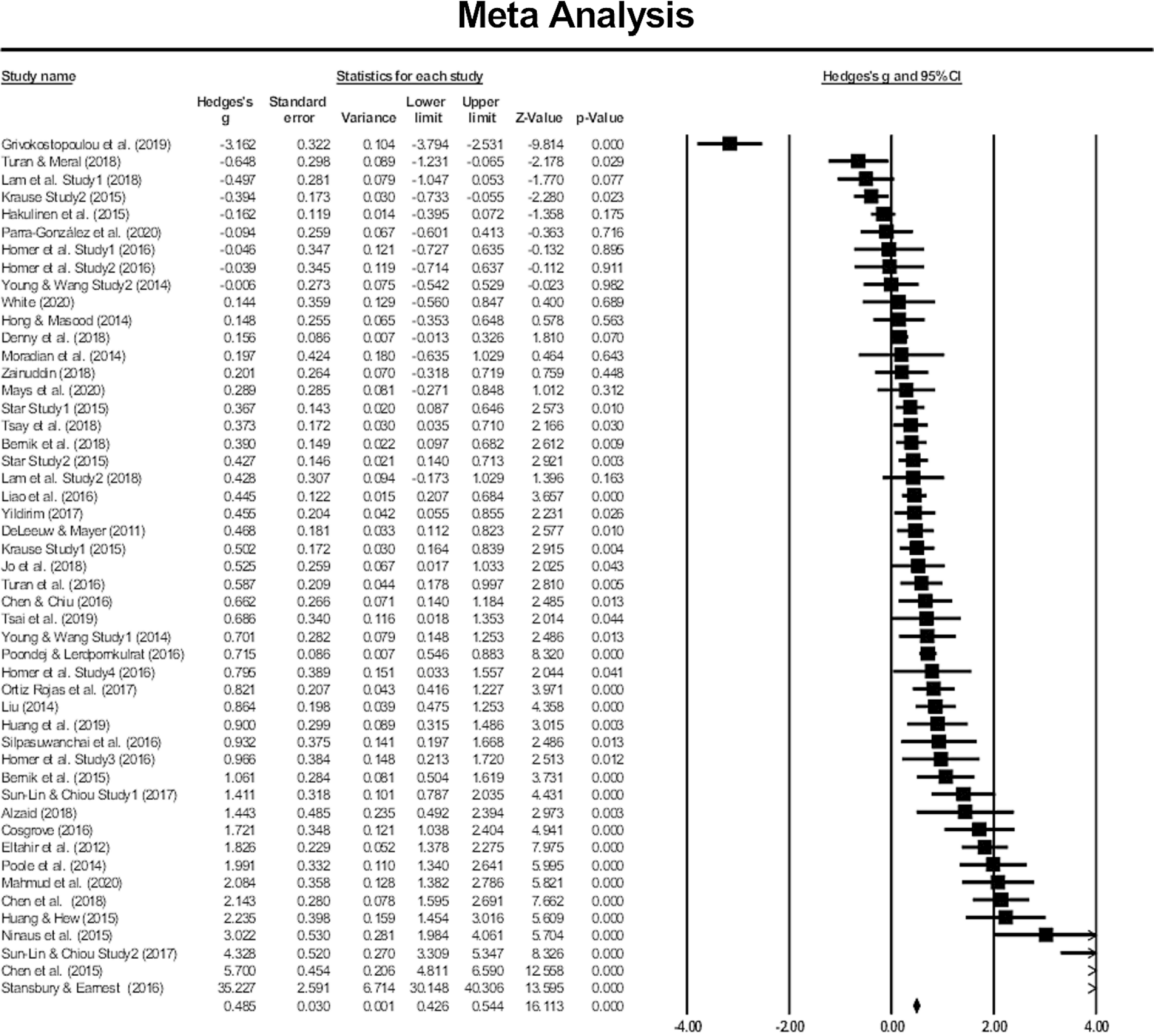
Forest plot of effect size (Hedges’ *g*) in a random-effects model.

Figure 2 displays a forest plot that presents 49 independent studies with a total of 5,071 participants along with their author(s), publication year, Hedge’s *g* (organized by effect size), standard error, variance, confidence interval, *Z*-value, and value of *p*. The random-effects model yielded an overall effect size of *g* = 0.822 (95%CI [0.567–1.078], *p* < 0.001), indicating a statistically significant effect size (Cohen, 1992).

The *Q*-statistic is a statistical measure used to evaluate whether all the studies analyzed share a common effect size (Borenstein et al., 2021). In this study, the *Q*-value was calculated at 812.417 with 48 degrees of freedom and a value of p of *p* < 0.001. Consequently, the null hypothesis that the effect size is identical across all studies is rejected. Additionally, *T*^2^ was measured at 0.730 and *I*^2^ at 94. 092, indicating that 95. 1% of the observed heterogeneity observed can be attributed to between-study differences, with only 4.9% resulting from sampling error. As a result, the moderator analysis that followed was deemed justifiable (Borenstein et al., 2021).

### 3.2. Moderator analysis

Moderator analysis was conducted to investigate whether the variables of interest moderated the impact of gamification on learning outcomes. Table 2 displays the results of the moderator analysis performed on six categorical variables. Five variables (i.e., user type, educational discipline, design principles for educational gamification, duration of “gameful” experience, and learning environment) had a significant moderating effect. In contrast, the moderating effects of the publication type were found to be weak.

**Table 2:** Moderator analysis.

Moderator variable	Effect size and 95% confidence interval					Heterogeneity	
	*k*	*g*	*SE*	Lower	Upper	*Q*-value	*p*
User type							
Elementary school user	12	1.293	0.379	0.550	2.035	21.126**	0.000
Secondary school user	29	0.869	0.163	0.550	1.188		
Higher education user	8	0.014	0.141	−0.263	0.291		
Educational discipline							
Science	6	3.220	0.799	1.654	4.786	19.052**	0.004
Math	3	2.005	0.930	0.181	3.828		
Engineering/computing	13	0.998	0.246	0.516	1.480		
Social science	16	0.472	0.150	0.179	0.765		
Business	5	0.031	0.591	−1.128	1.189		
Others	1	0.144	0.359	−0.560	0.847		
Not mentioned	5	0.769	0.357	0.070	1.468		
Design principles for educational gamification							
Dynamics	1	0.445	0.122	0.070	1.468	19.052**	0.000
Dynamics + Esthetics	1	−3.162	0.322	−1.128	1.189		
Mechanics	15	0.533	0.178	0.516	1.480		
Mechanics + Dynamics	17	0.997	0.202	0.181	3.828		
Mechanics + Dynamics + Esthetics	15	1.285	0.292	−0.560	0.847		
Duration of “gameful” experience							
<1 week	5	1.874	0.866	0.176	3.571	15.512**	0.008
1 week–1 month	15	0.621	0.294	0.044	1.198		
1 month–3 months	14	0.519	0.144	0.237	0.801		
3 months–1 semester	6	0.480	0.204	0.080	0.880		
>1 semester	5	3.304	0.794	1.747	4.860		
Not mentioned	4	0.968	0.342	0.298	1.638		
Learning environment							
Online	12	0.340	0.241	−0.131	0.812	180.408**	0.000
Hybrid	36	0.863	0.127	0.613	1.112		
Offline	1	35.227	2.591	30.148	40.306		
Measurement of student outcomes						8.416	0.015
Academic performance	30	1.015	0.216	0.592	1.438		
Engagement	16	0.383	0.116	0.155	0.610		
Motivation	3	2.206	1.263	−0.270	4.682		
Publication type							
Journal article	35	0.936	0.185	0.573	1.299	1.841	0.389
Thesis/dissertation	5	0.722	0.234	0.264	1.179		
Conference proceeding	9	0.585	0.182	0.227	0.942		

*k* = number of studies; 95% CI = 95% confidence interval; Q = total variability; **p < 0.01.

#### 3.2.1. User type

Our analysis revealed statistically significant moderation effects for user type (*Q*_*between*_ = 21.126, *p* = 0.000). *Post-hoc* comparisons showed that the effect size of elementary school learners (Hedges’ *g* = 1.293) was significantly larger than that of secondary school learners (Hedges’ *g* = 0.014, *Q*_*between*_ = 10.010, *p* = 0.002). Similarly, higher education users (Hedges’ *g* = 0.869) had a significantly higher effect size compared to secondary school users (Hedges’ *g* = 0.014, *Q*_*between*_ = 15.757, *p* = 0.000).

#### 3.2.2. Educational discipline

The variable discipline also had a significant moderating effect. Studies implemented in the subject area of science showed the strongest effect size (Hedges’ *g* = 3.220), followed by math (Hedges’ *g* = 2.005), engineering/computing (Hedges’ *g* = 0.998), social science (Hedges’ *g* = 0.472), and business (Hedges’ *g* = 0.031). Pairwise comparisons suggested that the effect size in science was significantly higher than in the subject domain of business (*Q*_*between*_ = 10.297, *p* = 0.001), engineering/computing (*Q*_*between*_ = 7.065, *p* = 0.008), social science (*Q*_*between*_ = 11.428, *p* = 0.001).

#### 3.2.3. Design principles for educational gamification

The moderating role of design principles for educational gamification on the association between gamification and learning outcomes was found to be statistically significant (*Q*_*between*_ = 19.052, *p* = 0.004). The effect size of the “dynamics + esthetics” subcategory was negative (Hedges’ *g* = −3.162), whereas the effect sizes of the other four subcategories were positive. Further *post-hoc* analysis suggested that the effect size of “dynamics + esthetics” (Hedges’ *g* = −3.162) was significantly different from those of the other subcategories, with the largest effect size seen in the “mechanics + dynamics + esthetics” subcategory (Hedges’ *g* = 1.285). Additionally, we found a significant difference in effect size between “dynamics” (Hedges’ *g* = 0.445) and “dynamics + esthetics + mechanics” (Hedges’ *g* = 1.285, *Q*_*between*_ = 7.024, *p* = 0.008).

#### 3.2.4. Duration of “gameful” experience

Moderation analysis indicated significant differences in the duration of “gameful” experience (*Q*_*between*_ = 15.512, *p* = 0.008). *Post-hoc* analysis revealed that the effect size for the “gameful” experience lasting “> one semester” (Hedges’ *g* = 3.304) was significantly larger than that of the “gameful” experience lasting “1 month-3 months” (Hedges’ *g* = 0.519, *Q*_*between*_ = 11.908, *p* = 0.001), “1 week-1 month” (Hedges’ *g* = 0.621, *Q*_*between*_ = 10.033, *p* = 0.002), and “3 months-1 semester” (Hedges’ *g* = 0.480, *Q*_*between*_ = 11.857, *p* = 0.001). These results suggest that the “gameful” experience lasting more than one semester has a significantly greater impact on learning outcomes than shorter interventions.

#### 3.2.5. Learning environment

The moderator analysis indicated a significant relationship between the learning environment and the effect of gamification on academic achievement (*Q*_*between*_ = 180.408, *p* = 0.000). *Post-hoc* pairwise comparisons showed that the “offline” condition (Hedges’ *g* = 35.227) had a statistically significant difference compared to both the “online” (Hedges’ *g* = 0.340, *Q*_*between*_ = 179.723, *p* = 0.000) and “hybrid” (Hedges’ *g* = 0.863, *Q*_*between*_ = 175.462, *p* = 0.000) conditions.

#### 3.2.6. Measurement of student outcomes

Moderation analysis showed no statistically significant differences between the different measures of student outcomes (Hedges’ *g* = 8.416, *p* = 0.015). Among the measures examined, motivation had the largest effect size (Hedges’ *g* = 2.206), followed by academic performance (Hedges’ *g* = 1.015) and engagement (Hedges’ *g* = 0.383).

#### 3.2.7. Publication type

Our analysis revealed no significant variation between the different types of publications (Hedges’ *g* = 1.841, *p* = 0.389). In fact, the journal article reported the largest effect size (Hedges’ *g* = 0.936), followed by the thesis/dissertation (Hedges’ *g* = 0.722) and the conference proceeding (Hedges’ *g* = 0.585).

### 3.3. Publication bias

To obtain an accurate measurement of the effect size in a particular field, a meta-analysis must incorporate a sample of studies that is representative of that field. Nevertheless, if a meta-analysis only includes a biased selection, the reported effect size may become distorted. This type of sampling issue is commonly referred to as publication bias.

**Figure 3:**
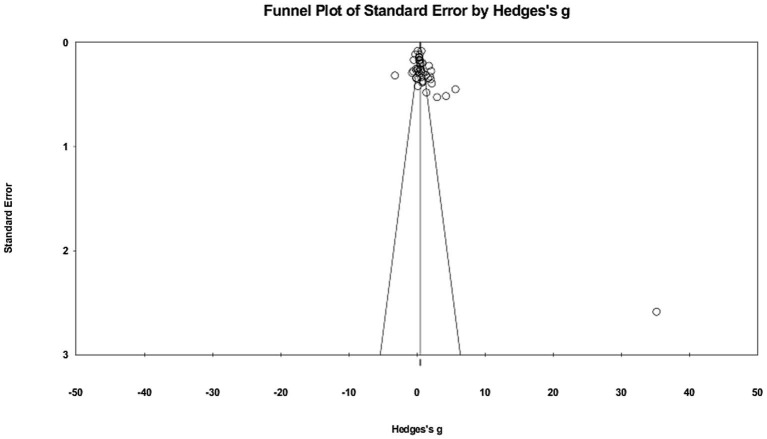
Funnel plot of effect size data.

A funnel plot was utilized to estimate publication bias, which is a widely used method for detecting such bias (Borenstein et al., 2021). The funnel plot in Figure 3 presents a generally symmetrical dispersion around the weighted mean effect sizes. Funnel plots display effect sizes calculated from studies included in a meta-analysis plotted against the standard error (Sterne and Egger, 2001). The horizontal axis represents Hedge’s, with the standard error plotted on the vertical axis. The funnel plot is considered to be symmetrical in the absence of publication bias, but visual inspection is not considered a definitive method (Duval and Tweedie, 2000).

Along with the funnel plot, the fail-safe N test was also performed. The fail-safe N test revealed that a substantial number, 4901, of additional studies would have to be conducted to counterbalance the overall effect size calculated in the current meta-analysis. Since it was unlikely that a substantial number of studies were neglected, together with the results of the funnel plot, we believe that publication bias is unlikely to be an issue in this meta-analytical research.

## 4. Discussion

Implementing gamification in educational settings can be a complicated endeavor, requiring collaboration and resources from various professionals. Therefore, it is crucial to examine the effectiveness of this instructional approach and understand which design features are effective under different circumstances. The current meta-analysis focused on student learning outcomes in gamification studies to shed light on the effectiveness of gamification and the moderators that improve learning outcomes, contributing to optimizing its use in educational settings.

To achieve this, this meta-analysis had two main objectives: (a) to estimate the overall effect size of gamification on learning outcomes, and (b) to identify factors contributing to variations in the effect sizes across studies through moderator analysis. Our results indicated a large effect size (*g* = 0.822 [0.567 to 1.078]) for the first objective. This effect size is much higher than that reported in prior research (0.40–0.60) (Bai et al., 2020; Huang et al., 2020; Sailer and Homner, 2020), providing strong evidence to support the use of gamification as an instructional approach in educational contexts.

For the second objective, the moderator analysis has shed light on the potential factors that may impact the magnitude of the effect. Regarding the user types, a significant moderating effect was found (*Q*_*between*_ = 21.126, *p* = 0.000), suggesting the effectiveness of gamification on student achievement varies across user types. Primary school users yielded a significantly larger effect size than secondary school users (*Q*_*between*_ = 10.010, *p* = 0.002). One possible reason could be related to differences in their motivation levels. Primary school learners may be more intrinsically motivated to learn (Vygotsky, 2016), and less focused on external rewards, which could enhance their engagement with gamification activities and lead to better academic achievement. In contrast, secondary learners may be more extrinsically motivated, and more focused on grades, which could limit their engagement with gamification activities and thus contribute to a lower effect size. Another possible explanation is that primary school users may have fewer preconceptions about traditional teaching methods and be more open to alternative approaches (e.g., gamified approaches) learning (Murillo and Martinez-Garrido, 2014). Furthermore, users at the higher education level exhibited a significantly larger effect size than secondary school users (*Q*_*between*_ = 15.757, *p* = 0.000). This difference could be attributed to their higher intrinsic motivation to learn, which gamification activities can sustain over the long term. Another possible explanation concerns the greater familiarity of higher education users with technology and digital tools (Kim and Castelli, 2021), which makes them more receptive to gamified learning activities that incorporate these resources. This familiarity can also make it easier for them to engage with the various features and functions of gamified learning activities, which may be more complex or multifaceted than those used in secondary school contexts.

The educational discipline in which gamification is applied significantly impacts its effectiveness in promoting student achievement. Science showed the strongest effect size, followed by math, engineering/computing, social science, and business, with significant differences identified between science and business (*Q*_*between*_ = 10.297, *p* = 0.001), engineering/computing (*Q*_*between*_ = 7.065, *p* = 0.008), and social science (*Q*_*between*_ = 11.428, *p* = 0.001). These results are consistent with previous studies that have demonstrated the effectiveness of gamification in science education (Kapp, 2012; Chen et al., 2018). Several potential factors may contribute to the variation across different subject disciplines. One possible reason is the nature of the subject matter. Science education is generally known to involve complex concepts and problem-solving tasks that may be more challenging for students to grasp (Hmelo-Silver, 2004), making gamification an effective way to increase engagement and motivation. In addition, hands-on learning opportunities in science education may lend themselves well to gamification activities. Pedagogical approaches may also play a part, with inquiry-based and problem-based learning commonly used in science education aligning well with gamified learning (Lai and Bower, 2020). In contrast, business education may be more lecture-based and centered on theoretical knowledge (Carriger, 2015), making it less suitable for gamified learning approaches that rely on exploration and experimentation. These findings may have practical implications for instructional designers and educators, who should consider the subject discipline when designing and utilizing gamification in educational contexts.

The current meta-analysis provides evidence that design principles for educational gamification can be effective for improved learning outcomes in educational contexts. However, the effectiveness of gamification seems to be contingent on the particular design principle. The highest effect size (Hedges’ *g* = 1.285) was seen in the “mechanics + dynamics + esthetics” subcategory, suggesting that combining three may be the most effective design principle for promoting gamified learning outcomes. The possible reason for this could be that such a combination offers a well-rounded approach that caters to both the measurable and intangible aspects of the gaming experience while also addressing subjective factors. Mechanics are defined as the quantifiable components of the game (e.g., rewards, prizes, points, and rankings). These elements are concrete and can be easily measured and tracked. Dynamics, on the other hand, designates to the behavior of the game mechanics over time and how they interact with each other and with the player (Hakulinen et al., 2013). Dynamics are the intangible aspects of the game that can create a unique experience for each player (e.g., events, tasks, feedback, and competition) (Hakulinen et al., 2013). Esthetics are the emotional responses that players experience when engaging with the game system, which may encompass elements of sensations, narrative, companionship, expression, or entertainment (Hakulinen et al., 2013). Our finding aligns with the MDA framework, which underscores the significance of the cohesive interplay between all three components in generating successful learning outcomes. This may be due to the clear goals and feedback provided by mechanics, as well as the engaging and immersive experience created by esthetics. Moreover, the observed difference in effect size between the combination of three and “dynamics” suggests that including mechanics and esthetics in gamified design may result in better learning outcomes. This could be attributed to mechanics offering explicit goals and feedback for the player, while esthetics contributes to a captivating and immersive game experience. Conversely, the combination of dynamics and esthetics alone may hinder the effectiveness of gamification concerning the identified negative effect size (Hedges’ *g* = −3.162). The possible explanations for this adverse effect size may pertain to the working mechanism of the MDA framework (Hakulinen et al., 2013). From the designers’ perspective, the MDA framework suggests that game mechanics should be established before moving on to dynamics and esthetics. This sequence guarantees that the structure of game elements is grounded in rules and systems before attending to the subjective aspects of player experience. Therefore, the negative effect size of “dynamics + esthetics” could be ascribed to the lack of clear goals and feedback provided by the mechanics, resulting in reduced learning outcomes. On the other hand, from the users’ perspective, esthetics may be the first aspect of the gamified learning they encounter, followed by dynamics and then mechanics. This sequence implies that learners may prioritize the experiential aspects of the game elements (e.g., immersion, engagement) over the more concrete elements (e.g., goals, feedback). Thus, if the mechanics fail to captivate the learners, they might not be motivated to persist in playing and learning, leading to a negative effect size for “dynamics + esthetics.”

The duration of “gameful” experience was revealed to be a significant moderator (*Q*_*between*_ = 15.512, *p* = 0.008). The duration of “gameful” experience lasting “>1 semester” were found to have a significantly larger effect size than gamified interventions lasting “1 month-3 months,” “1 week-1 month,” and “3 months-1 semester”. The findings add to the existing literature (Beemer et al., 2019; Sailer and Homner, 2020) on the duration of “gameful” experience, highlighting the importance of longer-term interventions in enhancing learning outcomes. Several potential causes may contribute to these findings. First, longer “gameful” experiences may give students more time to consolidate their learning, leading to higher retention of knowledge and improved learning outcomes. Moreover, prolonged exposure to the game mechanics in longer interventions may promote a deeper understanding of the learning content. As learners become more proficient with the game mechanics, they may be more capable of concentrating on the learning material and achieving better performance. Another possible explanation for the larger effect size observed in longer “gameful” experiences is that they allow learners to explore diverse strategies and techniques for navigating the game mechanics. With more time devoted to practice, learners may be able to more effectively apply their knowledge to different situations, resulting in improved learning outcomes. It is worth noting that the “gameful” experience lasting “<1 week” did not have a statistically significant effect size compared to other types. However, it still had a larger effect size than interventions lasting “1 month-3 months,” “1 week-1 month,” and “3 months-1 semester.” This finding may be ascribed to the phenomenon of hyperbolic discounting, which refers to people’s cognitive tendency to prefer short-term rewards over long-term rewards (Ainslie, 1975; Standage et al., 2005; Kim and Castelli, 2021).

Regarding the learning environment, our analysis revealed statistically significant moderation effects (*Q*_*between*_ = 180.408, *p* = 0.000), indicating its importance in the effectiveness of gamification in educational settings. The offline learning environment was found to have a significantly greater effect size compared to both online and hybrid learning environments. Three explanations may account for this unexpected outcome. The first one pertains to tangible learning experiences. Offline learning environments may offer more hands-on or tangible learning experiences (e.g., awarding real gifts or badges), which can better engage and motivate learners and improve their performance (Çakıroğlu et al., 2017). Second, personalized feedback may play a role. The “offline” condition may offer more opportunities for learners to receive personalized feedback and guidance from instructors. At the same time, digital learning environments may provide only automated feedback without a detailed explanation, often not tailored to individual needs. According to Ebadi et al. (2021), technical delays and the high pace of the game are among the most frequently reported issues in digital learning, which may lead to learner demotivation and poor performance. Third, social interaction may possibly contribute to the variation. Offline learning environments allow for more face-to-face interaction and collaboration among learners, leading to improved learning outcomes. Online education, on the other hand, may leave students feeling isolated from instructors and peers, which can hinder their learning achievement (Manner, 2003). In addition, online support groups are continuously open to new membership and may experience fluctuations in membership, making it difficult for them to engage in effective group work and ultimately leading to lower performance (Gary and Remolino, 2000). Therefore, it is essential for educators or instructors to carefully design the learning environment to maximize the effectiveness of gamification in promoting academic achievement.

In relation to the measurement of student outcomes, no significant moderating effect was observed (*Q*_*between*_ = 8.416, *p* = 0.015). This implies that the effectiveness of gamification in educational settings remains consistent across different measures employed to assess student outcomes. However, there were variances in effect sizes observed among these measures. Among them, motivation showed the largest effect size (Hedges’ *g* = 2.206). There are several potential reasons that could account for this finding. Firstly, gamification has the capacity to create an environment that allows for diverse and divergent thinking, leading to the exchange of multiple perspectives among students. Such an environment promotes collective learning and collaboration, thereby enhancing intrinsic motivation (Stansbury and Earnest, 2016). Secondly, the social aspect of gamified environments can also contribute to increased motivation. Many gamifications incorporate elements of competition and collaboration, allowing students to compete against each other or work together towards a common goal. This social interaction and sense of community can foster a sense of belonging and purpose, which are essential drivers of motivation (Buckley and Doyle, 2016; Koivisto and Hamari, 2019). Furthermore, the use of game elements in gamified environments also serves as a motivational affordance, simulating learners’ learning behavior (Inocencio, 2018). By incorporating game mechanics into the educational experience, gamification may tap into individuals’ intrinsic motivation to achieve goals and overcome challenges (Manzano-León et al., 2021). These elements create a sense of accomplishment and progress, which can be highly motivating for students. On the other hand, the weakest effect size was observed for engagement (Hedges’ *g* = 0.383), potentially due to learners’ perceived value of education. González-Fernández et al. (2022) suggested that learners’ behavioral engagement can be influenced by the importance they attach to their education. If gamification elements fail to address and encourage these factors sufficiently, it can negatively impact the observed levels of engagement. Therefore, game elements should be carefully designed to promote positive engagement and tailored to users’ needs and preferences, underscoring the necessity for more rigorous primary study designs to effectively mitigate alternative explanations for variations in learning outcomes across varying conditions (Sailer and Homner, 2020).

Concerning publication type, no significant moderating effect was observed (*Q*_*between*_ = 1.841, *p* = 0.389). However, we observed variations in effect size across different kinds of publications. Specifically, journal articles had the largest effect size (Hedges’ *g* = 0.936), followed by theses/dissertations (Hedges’ *g* = 0.722), and conference proceedings had the smallest effect size (Hedges’ *g* = 0.585). While these differences were not statistically significant, they may be indicative of underlying factors that influenced the effectiveness of gamification in different types of studies. One possible explanation for the observed differences in effect size is that certain types of publications may be more likely to include rigorous research designs and methods, which could lead to larger effect sizes. For example, journal articles typically undergo a rigorous peer-review process and are held to high standards of research quality and validity. The findings may suggest that the quality and rigor of the research may play a role in determining the effectiveness of gamification in educational contexts. Besides, further research is needed to fully understand the factors that influence the effectiveness of gamification in different types of publications.

## 5. Limitations

There are several limitations to be considered in this research. Firstly, it is important to acknowledge the possibility of variability in the quality and reliability of the included studies. Despite extensive efforts to adhere to stringent inclusion criteria and methodological rigor, inherent disparities within the study design or sample sizes among the selected studies may persist. These variations could have the potential to introduce heterogeneity into the analysis and may possibly affect the generalizability of results to some extent. Nonetheless, we made a concerted effort to carefully consider and address these potential variations in study characteristics within the scope of this research.

Additionally, this study investigated the moderating effect of several important variables based on a comprehensive review of the literature. However, given the relative novelty and rapid evolution of the field of gamification, we also identified additional emerging variables that could potentially impact gamification research and represent valuable avenues for further investigation. These variables include player characteristics (Van Berlo et al., 2023), the role of user experience (Anim et al., 2023), reliability of outcome measures (Inocencio, 2018), teaching role of the family (Gözüm and Kandır, 2020, 2021; Papadakis et al., 2022). Unfortunately, despite thoroughly examining and coding variables from the identified studies, we encountered challenges in obtaining sufficient data that met the necessary inclusion and exclusion criteria. Consequently, we were unable to include these variables in our analysis. It is our hope that future empirical investigations in related areas will consider yielding the requisite data to enable their incorporation into future meta-analyses.

## 6. Conclusion and implications

This study has produced valuable findings regarding the impact of gamification on student learning outcomes, which may contribute to advancing our knowledge of its potential in educational contexts. First, our study revealed a significant overall effect size of 0.822 [0.567 to 1.078] for gamification on student learning outcomes, implying that gamification may hold promise as a viable approach for promoting teaching and learning in diverse educational contexts. Second, we discovered that several factors had a moderating impact, including the user type (e.g., primary school users demonstrated the greatest effect size), educational discipline (e.g., science students exhibited a considerably higher effect size than those in the disciplines of business, engineering/computing, and social science), design principles for educational gamification (e.g., the largest effect size observed in the design principle of “mechanics + dynamics + esthetics”), duration of “gameful” experience (e.g., the gamified interventions lasting more than one semester showed the greatest impact), and learning environment (e.g., the “offline” condition produced the largest effect size). These findings aid researchers in pinpointing the variables that impact gamification’s effectiveness in improving learning outcomes. We expect these results will encourage greater interest in gamification research and more inquiry into its potential for enhancing teaching and learning in educational contexts.

The findings of this study provide significant insights into the use of gamification to enhance student learning outcomes. Based on the results, the following recommendations are proposed to optimize the effectiveness of gamification in educational contexts:

1. The “gameful” experience should last more than one semester to produce significant gains in educational outcomes.
2. Consideration should be given to the design principles for educational gamification as certain types of design principles have been found to be more effective than others. Specifically, the “mechanics + dynamics + esthetics” sub-category had the largest effect size and is highly recommended. However, the “dynamics + esthetics” subcategory had a negative effect size and should be avoided in the design and implementation of gamification.
3. The educational discipline in which gamification is implemented should also be considered. Studies implemented in the subject area of science showed the strongest effect size.
4. The learning environment in which gamification is implemented can also have a significant impact on academic achievement. The offline condition had the largest effect size compared to the online and hybrid conditions. However, as a limited number of empirical studies included in this analysis examined the offline learning environment, further research is recommended to confirm our findings.
5. The user type of the students should also be considered. Elementary and higher education users had a significantly higher effect size compared to secondary school users. Therefore, it is advisable to consider the appropriateness of gamification for users of different educational levels carefully and to tailor its design and implementation accordingly.
6. While the measurement of student outcomes did not yield any significant differences, motivation was found to have the largest effect size, while engagement had the least. To optimize gamification in education, it is suggested to focus on motivation by integrating features that boost intrinsic motivation, foster collaboration, and stimulate healthy competition. It is crucial to tailor game elements to suit the learners’ individual needs and preferences, ensuring their engagement. To achieve dependable and valid assessments of learning outcomes, a rigorous study design must be implemented.
7. Lastly, while there were no significant differences between types of publications, journal articles reported the largest effect size. These variations in effect size across different types of publications may suggest that the quality and rigor of the research design and methods play a crucial role in determining the effectiveness of gamification in educational contexts. Therefore, it is recommended to employ rigorous research designs and methods to ensure high research quality and validity.

## Data availability statement

The original contributions presented in the study are included in the article/supplementary material, further inquiries can be directed to the corresponding authors.

## Author contributions

ML: Conceptualization, Data curation, Formal analysis, Funding acquisition, Investigation, Methodology, Project administration, Software, Supervision, Writing – original draft, Writing – review & editing. SM: Conceptualization, Data curation, Investigation, Resources, Supervision, Validation, Visualization, Writing – review & editing, Software. YS: Project administration, Resources, Validation, Visualization, Writing – review & editing, Investigation, Conceptualization, Software.

## References

[ref1] Ainslie G. (1975). Specious reward: a behavioral theory of impulsiveness and impulse control. Psychol. Bull. 82, 463–496. doi: 10.1037/h0076860, PMID: 1099599

[ref2] Alsawaier R. S. (2018). The effect of gamification on motivation and engagement. Int. J. Inf. Learn. Technol. 35, 56–79. doi: 10.1108/ijilt-02-2017-0009

[ref3] Alzaid F. (2018). The effects of gamification based formative assessment on motivation and vocabulary acquisition in ESL classroom. Montreal, Quebec, Canada: McGill University.

[ref4] Anim N. A. H. M. Suffarruddin S. H. Najib N. M. Sabarudin N. A. (2023). Ensuring the sustainability of sadaqah based crowdfunding platforms: the role of gamification and user experience. J. Muamalat Islam. Finan. Res. 20, 35–48. doi: 10.33102/jmifr.475

[ref5] Bai S. Hew K. F. Huang B. (2020). Does gamification improve student learning outcome? Evidence from a meta-analysis and synthesis of qualitative data in educational contexts. Educ. Res. Rev. 30:100322. doi: 10.1016/j.edurev.2020.100322

[ref6] Beemer L. R. Ajibewa T. A. DellaVecchia G. Hasson R. E. (2019). A pilot intervention using gamification to enhance student participation in classroom activity breaks. Int. J. Environ. Res. Public Health 16:4082. doi: 10.3390/ijerph16214082, PMID: 31652885 PMC6862043

[ref7] Bernik A. Bubaš G. Radošević D. (2015). A pilot study of the influence of gamification on the effectiveness of an e-learning course. In Central European conference on information and intelligent systems.

[ref8] Bernik A. Bubaš G. Radošević D. (2018). Measurement of the effects of e-learning courses gamification on motivation and satisfaction of students. In 2018 41st International Convention on Information and Communication Technology, Electronics and Microelectronics (MIPRO).

[ref9] Borenstein M. Hedges L. Higgins J. Rothstein H. (2005). Comprehensive meta-analysis (computer program) version 2. Englewood, NJ: Biostat.

[ref10] Borenstein M. Hedges L. V. Higgins J. P. Rothstein H. R. (2021). Introduction to meta-analysis. Hoboken, New Jersey, U.S: John Wiley & Sons.

[ref11] Brophy S. Cooksey R. Davies H. Dennis M. S. Zhou S. M. Siebert S. (2013). The effect of physical activity and motivation on function in ankylosing spondylitis: a cohort study. Semin. Arthritis Rheuma. 42, 619–626. doi: 10.1016/j.semarthrit.2012.09.007 PMC3685805 23351615

[ref12] Buckley P. Doyle E. (2016). Gamification and student motivation. Interact. Learn. Environ. 24, 1162–1175. doi: 10.1080/10494820.2014.964263

[ref13] Çakıroğlu Ü. Başıbüyük B. Güler M. Atabay M. Memiş B. Y. (2017). Gamifying an ICT course: influences on engagement and academic performance. Comput. Hum. Behav. 69, 98–107. doi: 10.1016/j.chb.2016.12.018

[ref14] Caponetto I. Earp J. Ott M. (2014). Gamification and education: a literature review. In European Conference on Games Based Learning Academic Conferences International Limited.

[ref15] Carriger M. S. (2015). Problem-based learning and management development–empirical and theoretical considerations. Int. J. Manag. Educ. 13, 249–259. doi: 10.1016/j.ijme.2015.07.003

[ref16] Charness G. Gneezy U. Kuhn M. A. (2012). Experimental methods: between-subject and within-subject design. J. Econ. Behav. Organ. 81, 1–8. doi: 10.1016/j.jebo.2011.08.009

[ref17] Chen C. H. Chiu C. H. (2016). Employing intergroup competition in multitouch design-based learning to foster student engagement, learning achievement, and creativity. Comput. Educ. 103, 99–113. doi: 10.1016/j.compedu.2016.09.007

[ref18] Chen C.-C. Huang C. Gribbins M. Swan K. (2018). Gamify online courses with tools built into your learning management system (LMS) to enhance self-determined and active learning. Online Learn. J. 22, 41–54. doi: 10.24059/olj.v22i3.1466

[ref19] Chen C. H. Liu G. Z. Hwang G. J. (2015). Interaction between gaming and multistage guiding strategies on students’ field trip mobile learning performance and motivation. Br. J. Educ. Technol. 47, 1032–1050. doi: 10.1111/bjet.12270

[ref20] Cohen J. (1992). A power primer. Psychol. Bull. 112, 155–159. doi: 10.1037/0033-2909.112.1.155 19565683

[ref21] Cordero-Brito S. Mena J. (2020). Gamification and its application in the social environment: a tool for shaping behaviour. J. Inform. Technol. Res. 13, 58–79. doi: 10.4018/JITR.2020070104

[ref22] Cosgrove P.J. (2016). The effects of gamification on self-efficacy and persistence in virtual world familiarization. [Doctoral dissertation]. University of Missouri—Columbia.

[ref23] DeLeeuw K. E. Mayer R. E. (2011). Cognitive consequences of making computer-based learning activities. More game-like. Comput. Hum. Behav. 27, 2011–2016. doi: 10.1016/j.chb.2011.05.008

[ref24] Denny P. McDonald F. Empson R. Kelly P. Petersen A. (2018). Empirical support for a causal relationship between gamification and learning outcomes. In Proceedings of the 2018 CHI conference on human factors in computing systems.

[ref25] Deterding S. Dixon D. Khaled R. Nacke L. (2011). “From game design elements to Gamefulness: defining gamification.” In Proceedings of the 15th International Academic MindTrek Conference: Envisioning Future Media Environments. 9–15.

[ref26] Dichev C. Dicheva D. (2017). Gamifying education: what is known, what is believed and what remains uncertain: a critical review. Int. J. Educ. Technol. High. Educ. 14, 1–36. doi: 10.1186/s41239-017-0042-5

[ref27] Domínguez A. Saenz-de-Navarrete J. De-Marcos L. Fernández-Sanz L. Pagés C. Martínez-Herráiz J.-J. (2013). Gamifying learning experiences: practical implications and outcomes. Comput. Educ. 63, 380–392. doi: 10.1016/j.compedu.2012.12.020

[ref28] Duval S. Tweedie R. (2000). Trim and fill: a simple funnel-plot–based method of testing and adjusting for publication bias in meta-analysis. Biometrics 56, 455–463. doi: 10.1111/j.0006-341x.2000.00455.x, PMID: 10877304

[ref29] Ebadi S. Rasouli R. Mohamadi M. (2021). Exploring EFL learners’ perspectives on using Kahoot as a game-based student response system. Interact. Learn. Environ. 31, 2338–2350. doi: 10.1080/10494820.2021.1881798

[ref30] Educause. (2011). 7 things you should know about gamification. Available at: https://library.educause.edu/resources/2011/8/7-things-you-should-know-about-gamification (accessed on 10 April 2020)

[ref31] Eltahir M. E. Alsalhi N. R. Al-Qatawneh S. AlQudah H. A. Jaradat M. (2021). The impact of game-based learning (GBL) on students’ motivation, engagement and academic performance on an Arabic language grammar course in higher education. Educ. Inf. Technol. (Dordr). 26, 3251–3278. doi: 10.1007/s10639-020-10396-w

[ref32] Garland C. M. (2015). Gamification and implications for second language education: a meta- analysis. (Doctoral dissertation). St. Cloud State University, St. Cloud.

[ref33] Gary J. M. Remolino L. (2000). Coping with loss and grief through on-line support groups. ERIC/CASS Digest.

[ref34] González-Fernández A. Revuelta-Domínguez F. I. Fernández-Sánchez M. R. (2022). Models of instructional Design in Gamification: a systematic review of the literature. Educ. Sci. 12:44. doi: 10.3390/educsci12010044

[ref35] Gözüm A. İ. C. Kandır A. (2020). Developing a parental mediation scale of digital games for children. Int. J. Curric. Instruct. 12, 336–358.

[ref36] Gözüm A. İ. C. Kandır A. (2021). Digital games pre-schoolers play: parental mediation and examination of educational content. Educ. Inf. Technol. 26, 3293–3326. doi: 10.1007/s10639-020-10382-2

[ref37] Grivokostopoulou F. Kovas K. Perikos I. (2019). Examining the impact of a gamified entrepreneurship education framework in higher education. Sustainability 11:5623. doi: 10.3390/su11205623

[ref38] Groening C. Binnewies C. (2019). “Achievement unlocked!”-the impact of digital achievements as a gamification element on motivation and performance. Comput. Hum. Behav. 97, 151–166. doi: 10.1016/j.chb.2019.02.026

[ref39] Hakulinen L. Auvinen T. Korhonen A. (2013). Empirical study on the effect of achievement badges in TRAKLA2 online learning environment. In 2013 Learning and teaching in computing and engineering.

[ref40] Hmelo-Silver C. E. (2004). Problem-based learning: what and how do students learn? Educ. Psychol. Rev. 16, 235–266. doi: 10.1023/b:edpr.0000034022.16470.f3

[ref41] Homer R. Hew K. F. Tan C. Y. (2018). Comparing digital badges-and-points with classroom token systems: effects on elementary school ESL students’ classroom behavior and English learning. J. Educ. Technol. Soc. 21, 137–151.

[ref42] Hong G. Y. Masood M. (2014). Effects of gamification on lower secondary school students’ motivation and engagement. Int. J. Educ. Pedagog. Sci. 8, 3765–3772.

[ref43] Huang B. Hew K.F. (2015). Do points, badges and leaderboard increase learning and activity: a quasi-experiment on the effects of gamification. In Proceedings of the 23rd International Conference on Computers in Education.

[ref44] Huang B. Hew K. F. Lo C. K. (2019). Investigating the effects of gamification-enhanced flipped learning on undergraduate students’ behavioral and cognitive engagement. Interact. Learn. Environ. 27, 1106–1126. doi: 10.1080/10494820.2018.1495653

[ref45] Huang R. Ritzhaupt A. D. Sommer M. Zhu J. Stephen A. Valle N. . (2020). The impact of gamification in educational settings on student learning outcomes: a meta-analysis. Educ. Technol. Res. Dev. 68, 1875–1901. doi: 10.3102/1686773

[ref46] Hunicke R. LeBlanc M. Zubek R. (2004). MDA: a formal approach to game design and game research. In Proceedings of the AAAI Workshop on Challenges in Game.

[ref47] Inocencio F. (2018). Using gamification in education: a systematic literature review.

[ref48] Jacobs H. (2013). Gamification: a framework for the workplace. Liverpool: University of Liverpool.

[ref49] Jahnke J. (2010). Student perceptions of the impact of online discussion forum participation on learning outcomes. J. Learn. Des. 3, 27–34. doi: 10.5204/jld.v3i2.48

[ref50] Jo J. Jun H. Lim H. (2018). A comparative study on gamification of the flipped classroom in engineering education to enhance the effects of learning. Comput. Appl. Eng. Educ. 26, 1626–1640. doi: 10.1002/cae.21992

[ref51] Johnson D. Deterding S. Kuhn K. A. Staneva A. Stoyanov S. Hides L. (2016). Gamification for health and wellbeing: a systematic review of the literature. Internet Interv. 6, 89–106. doi: 10.1016/j.invent.2016.10.002, PMID: 30135818 PMC6096297

[ref52] Kapp K. M. (2012). The gamification of learning and instruction: Game-based methods and strategies for training and education. Hoboken, New Jersey, U.S: John Wiley & Sons.

[ref53] Kim J. Castelli D. M. (2021). Effects of gamification on behavioral change in education: a meta-analysis. Int. J. Environ. Res. Public Health 18:3550. doi: 10.3390/ijerph18073550, PMID: 33805530 PMC8037535

[ref54] Kiryakova G. Angelova N. Yordanova L. (2014). Gamification in education. In Proceedings of 9th international Balkan Education and Science Conference (1, 679–684).

[ref55] Koivisto J. Hamari J. (2019). The rise of motivational information systems: a review of gamification research. Int. J. Inf. Manag. 45, 191–210. doi: 10.1016/j.ijinfomgt.2018.10.013

[ref56] Krause M. Mogalle M. Pohl H. Williams J. J. (2015). A playful game changer: fostering student retention in online education with social gamification. In Proceedings of the Second (2015) ACM conference on Learning@ Scale.

[ref57] Lai J. W. Bower M. (2020). Evaluation of technology use in education: findings from a critical analysis. Of systematic literature reviews. J. Comput. Assist. Learn. 36, 241–259. doi: 10.1111/jcal.12412

[ref58] Lam Y. W. Hew K. F. Chiu K. F. (2018). Improving argumentative writing: effects of a blended learning approach and gamification. Lang. Learn. Technol. 22, 97–118. doi: 10.1007/978-981-287-089-6_5

[ref59] Landers R. N. (2014). Developing a theory of gamified learning: linking serious games and gamification of learning. Simul. Gaming 45, 752–768. doi: 10.1177/1046878114563660

[ref60] Lei H. Chiu M. M. Wang D. Wang C. Xie T. (2022). Effects of game-based learning on students’ achievement in science: a meta-analysis. J. Educ. Comput. Res. 60, 1373–1398. doi: 10.1177/07356331211064543

[ref61] Liao C. C. Y. Chang W. C. Chan T. W. (2018). The effects of participation, performance, and interest in a game-based writing environment. J. Comput. Assist. Learn. 34, 211–222. doi: 10.1111/jcal.12233

[ref62] Lipsey M. W. Wilson D. B. (2001). Practical meta-analysis. Washington DC: SAGE publications, Inc..

[ref63] Lister M. (2015). Gamification: the effect on student motivation and performance at the post-secondary level. ITLT. 3, 1–22. doi: 10.2458/azu_itet_v3i2_lister

[ref64] Liu T. Y. (2016). Using educational games and simulation software in a computer science course: learning achievements and student flow experiences. Interact. Learn. Environ. 24, 724–744. doi: 10.1080/10494820.2014.917109

[ref65] Lopez C. E. Tucker C. S. (2019). The effects of player type on performance: a gamification case study. Comput. Hum. Behav. 91, 333–345. doi: 10.1016/j.chb.2018.10.005

[ref66] Mahmud S. N. D. Husnin H. Tuan Soh T. M. (2020). Teaching presence in online gamified education for sustainability learning. Sustainability 12:3801. doi: 10.3390/su12093801

[ref67] Manner J. (2003). Avoiding esolation in online education. In Society for Information Technology & Teacher Education International Conference. in Association for the Advancement of computing in education (AACE), 408–410.

[ref68] Manzano-León A. Camacho-Lazarraga P. Guerrero M. A. Guerrero-Puerta L. Aguilar-Parra J. M. Trigueros R. . (2021). Between level up and game over: a systematic literature review of gamification in education. Sustainability 13:2247. doi: 10.3390/su13042247

[ref69] Marczewski A. (2013). Gamification: A simple introduction. Seattle: Amazon Digital Services, Inc.

[ref70] Mays B. R. Yeh H. C. Chen N. S. (2020). The effects of using audience response systems incorporating student-generated questions on EFL students’ reading comprehension. Asia-Pac. Educ. Res. 29, 553–566. doi: 10.1007/s40299-020-00506-0

[ref71] Mora A. Zaharias P. González C. Arnedo-Moreno J. (2016). Fraggle: a framework for agile gamification of learning experiences. In Games and Learning Alliance: 4th International Conference, GALA 2015, Rome, Italy, December 9–11, 2015, Revised Selected Papers 4 Springer International Publishing.

[ref72] Moradian A. Nasir M. Lyons K. Leung R. Sim S. E. (2014). “Gamification of collaborative idea generation and convergence.” in CHI’14 extended abstracts on human factors in computing systems.

[ref73] Morris M. G. Venkatesh V. (2000). Age differences in technology adoption decisions: implications for a changing workforce. Pers. Psychol. 53, 375–403. doi: 10.1111/j.1744-6570.2000.tb00206.x

[ref74] Murillo F. J. Martinez-Garrido C. (2014). Homework and primary-school students’ academic achievement in Latin America. Int. Rev. Educ. 60, 661–681. doi: 10.1007/s11159-014-9440-2

[ref75] Navarro-Mateos C. Pérez-López I. J. Marzo P. F. (2021). Gamification in the spanish educational field: a systematic review. Retos 42, 507–516. doi: 10.47197/retos.v42i0.87384

[ref76] Ninaus M. Pereira G. Stefitz R. Prada R. Paiva A. (2015). Game elements improve performance in a working memory training task. IJSG 2, 3–16. doi: 10.17083/ijsg.v2i1.60

[ref77] Ortiz-Rojas M.E. Chiluiza K. Valcke M. (2017). Gamification in computer programming: effects on learning, engagement, self-efficacy and intrinsic motivation. In 11th European Conference on Game-Based Learning (ECGBL).

[ref78] Page M. J. McKenzie J. E. Bossuyt P. M. Boutron I. Hoffmann T. C. Mulrow C. D. . (2021). The Prisma 2020 statement: an updated guideline for reporting systematic reviews. BMJ 372, 1–9. doi: 10.1136/bmj.n71 PMC8005924 33782057

[ref79] Papadakis S. Gözüm A. İ. C. Kalogiannakis M. Kandır A. (2022). “A comparison of Turkish and Greek parental mediation strategies for digital games for children during the COVID-19 pandemic,” In: STEM, robotics, mobile apps in early childhood and primary education. eds. Papadakis S. Kalogiannakis M. (Springer, Singapore: Lecture Notes in Educational Technology).

[ref80] Parra-González M. E. López Belmonte J. Segura-Robles A. Fuentes Cabrera A. (2020). Active and emerging methodologies for ubiquitous education: potentials of flipped learning and gamification. Sustainability 12:602. doi: 10.3390/su12020602 PMC7303505 32595557

[ref81] Poole S. M. Kemp E. Patterson L. Williams K. (2014). Get your head in the game: using gamification in business education to connect with generation Y. J. Bus. Excell. Edu. 3.

[ref82] Poondej C. Lerdpornkulrat T. (2016). The development of gamified learning activities to increase student engagement in learning. Aust. Educ. Comput. 31.

[ref83] Racey M. O’Brien C. Douglas S. Marquez O. Hendrie G. Newton G. (2016). Systematic review of school-based interventions to modify dietary behavior: does intervention intensity impact effectiveness? J. Sch. Health 86, 452–463. doi: 10.1111/josh.12396, PMID: 27122145

[ref84] Rachels J. R. Rockinson-Szapkiw A. J. (2018). The effects of a mobile gamification app on elementary students’ Spanish achievement and self-efficacy. Comput. Assist. Lang. Learn. 31, 72–89. doi: 10.1080/09588221.2017.1382536

[ref85] Ritzhaupt A. D. Huang R. Sommer M. Zhu J. Stephen A. Valle N. . (2021). A meta-analysis on the influence of gamification in formal educational settings on affective and behavioral outcomes. Educ. Technol. Res. Dev. 69, 2493–2522. doi: 10.1007/s11423-021-10036-1

[ref86] Rivera E. S. Garden C. L. P. (2021). Gamification for student engagement: a framework. J. Furth. High. Educ. 45, 999–1012. doi: 10.1080/0309877X.2021.1875201

[ref87] Sailer M. Hense J. U. Mayr S. K. Mandl H. (2017). How gamification motivates: an experimental study of the effects of specific game design elements on psychological need satisfaction. Comput. Hum. Behav. 69, 371–380. doi: 10.1016/j.chb.2016.12.033

[ref88] Sailer M. Homner L. (2020). The gamification of learning: a meta-analysis. Educ. Psychol. Rev. 32, 77–112. doi: 10.1007/s10648-019-09498-w

[ref89] Seaborn K. Fels D. I. (2015). Gamification in theory and action: a survey. Int. J. Hum. Comput. Stud. 74, 14–31. doi: 10.1016/j.ijhcs.2014.09.006

[ref90] Segura-Robles A. Fuentes-Cabrera A. Parra-González M. E. López-Belmonte J. (2020). Effects on personal factors through flipped learning and gamification as combined methodologies in secondary education. Front. Psychol. 11:1103. doi: 10.3389/fpsyg.2020.01103, PMID: 32595557 PMC7303505

[ref91] Silpasuwanchai C. Ma X. Shigemasu H. Ren X. (2016). Developing a comprehensive engagement of gamification for reflective learning. In Proceedings of the 2016 ACM Conference on Designing Interactive Systems.

[ref92] Standage M. Duda J. L. Ntoumanis N. (2005). A test of self-determination theory in school physical education. Br. J. Educ. Psychol. 75, 411–433. doi: 10.1348/000709904x22359 16238874

[ref93] Stansbury J. A. Earnest D. R. (2016). Meaningful gamification in an industrial/organizational psychology course. Psychol. Learn. Teach. 44, 38–45. doi: 10.1177/0098628316677645

[ref94] Star K. (2015). Gamification, interdependence, and the moderating effect of personality on performance. [Doctoral dissertation]. Coventry University.

[ref95] Sterne J. A. Egger M. (2001). Funnel plots for detecting bias in meta-analysis: guidelines on choice of axis. J. Clin. Epidemiol. 54, 1046–1055. doi: 10.1016/s0895-4356(01)00377-8 11576817

[ref96] Subhash S. Cudney E. A. (2018). Gamified learning in higher education: a systematic review of the literature. Comput. Hum. Behav. 87, 192–206. doi: 10.1016/j.chb.2018.05.028

[ref97] Sun-Lin H. Z. Chiou G. F. (2017). Effects of self-explanation and game-reward on sixth graders’ algebra variable learning. J. Educ. Technol. Soc. 13, 126–137. doi: 10.12973/eurasia.2017.01244a

[ref98] Tomaselli F. Sanchez O. Brown S. (2015). How to engage users through gamification: the prevalent effects of playing and mastering over competing.

[ref99] Tsai C. Y. Lin H. Liu S. C. (2020). The effect of pedagogical GAME model on students’ PISA scientific competencies. J. Comput. Assist. Learn. 36, 359–369. doi: 10.1111/jcal.12406

[ref100] Tsay C. H. H. Kofinas A. Luo J. (2018). Enhancing student learning experience with technology-mediated gamification: an empirical study. Comput. Educ. 121, 1–17. doi: 10.1016/j.compedu.2018.01.009

[ref101] Turan Z. Avinc Z. Kara K. Goktas Y. (2016). Gamification and education: achievements, cognitive loads, and views of students. Int. J. Emerg. Technol. Learn. 11, 64–69. doi: 10.3991/ijet.v11i07.5455

[ref102] Turan Z. Meral E. (2018). Game-based versus to non-game-based: the impact of student response systems on students’ achievements, engagements and test anxieties. Inform. Educ. 17, 105–116. doi: 10.15388/infedu.2018.07

[ref103] Van Berlo Z. M. C. Van Reijmersdal E. A. Waiguny M. K. J. (2023). Twenty years of research on gamified advertising: a systematic overview of theories and variables. Int. J. Advert. 42, 171–180. doi: 10.1080/02650487.2022.2143098

[ref104] Vygotsky L. S. (2016). Play and its role in the mental development of the child. Int. Res. Early Child. Educ. 5, 6–18. doi: 10.2753/rpo1061-040505036

[ref105] Wang Y.-S. Wu M.-C. Wang H.-Y. (2009). Investigating the determinants and age and gender differences in the acceptance of mobile learning. Br. J. Educ. Technol. 40, 92–118. doi: 10.1111/j.1467-8535.2007.00809.x

[ref106] Werbach K. Hunter D. (2015). The gamification toolkit: Dynamics, mechanics, and components for the win. Philadelphia: University of Pennsylvania Press.

[ref107] Werbach K. Hunter D. Dixon W. (2012). For the win: How game thinking can revolutionize your business (Vol. 1). Philadelphia: Wharton digital press.

[ref108] White N. (2020). Gamification, an instructional strategy to course design and impact on learning outcomes. [Doctoral dissertation]. Capella University.

[ref109] Wijaya T. T. Cao Y. Bernard M. Rahmadi I. F. Lavicza Z. Surjono H. D. (2022). Factors influencing microgame adoption among secondary school mathematics teachers supported by structural equation modelling-based research. Front. Psychol. 13:952549. doi: 10.3389/fpsyg.2022.952549, PMID: 36160545 PMC9493482

[ref110] Wongso O. Rosmansyah Y. Bandung Y. (2015). Gamification framework model, based on social engagement in e-learning 2.0. In Proceedings of 2014 2nd International Conference on Technology, Informatics, Management, Engineering and Environment, TIME-E 2014, pp. 10–14.

[ref111] Yıldırım I. (2017). The effects of gamification-based teaching practices on student achievement and students’ attitudes toward lessons. Internet High. Educ. 33, 86–92. doi: 10.1016/j.iheduc.2017.02.002

[ref112] Young S. S. C. Wang Y. H. (2014). The game embedded CALL system to facilitate English vocabulary acquisition and pronunciation. J. Educ. Technol. Soc. 17, 239–251.

[ref113] Zainuddin Z. (2018). Students’ learning performance and perceived motivation in gamified flipped-class instruction. Comput. Educ. 126, 75–88. doi: 10.1016/j.compedu.2018.07.003

[ref114] Zainuddin Z. Chu S. K. W. Shujahat M. Perera C. J. (2020). The impact of gamification on learning and instruction: a systematic review of empirical evidence. Educ. Res. Rev. 30:100326. doi: 10.1016/j.edurev.2020.100326

